# Author Correction: The role of manganese in CoMnO_x_ catalysts for selective long-chain hydrocarbon production via Fischer-Tropsch synthesis

**DOI:** 10.1038/s41467-025-56202-4

**Published:** 2025-01-20

**Authors:** Hao Chen, Zan Lian, Xiao Zhao, Jiawei Wan, Priscilla F. Pieters, Judit Oliver-Meseguer, Ji Yang, Elzbieta Pach, Sophie Carenco, Laureline Treps, Nikos Liakakos, Yu Shan, Virginia Altoe, Ed Wong, Zengqing Zhuo, Feipeng Yang, Ji Su, Jinghua Guo, Monika Blum, Saul H. Lapidus, Adrian Hunt, Iradwikanari Waluyo, Hirohito Ogasawara, Haimei Zheng, Peidong Yang, Alexis T. Bell, Núria López, Miquel Salmeron

**Affiliations:** 1 Chemical Sciences Division, Lawrence Berkeley National Laboratory, Berkeley, CA USA; 2 Institute of Chemical Research of Catalonia (ICIQ-CERCA), Barcelona Institute of Science and Technology (BIST), Tarragona, Spain; 3 Materials Science Division, Lawrence Berkeley National Laboratory, Berkeley, CA USA; 4 Department of Materials Science and Engineering, University of California, Berkeley, CA USA; 5 Department of Chemistry, University of California, Berkeley, CA USA; 6 Molecular Foundry, Lawrence Berkeley National Laboratory, Berkeley, CA USA; 7 Advanced Light Source, Lawrence Berkeley National Laboratory, Berkeley, CA USA; 8 Advanced Photon Source, Argonne National Laboratory, Lemont, IL USA; 9 National Synchrotron Light Source II, Brookhaven National Laboratory, Upton, NY USA; 10 SLAC National Accelerator Laboratory, Menlo Park, CA USA; 11 Department of Chemical and Biomolecular Engineering, University of California, Berkeley, CA USA

**Keywords:** Heterogeneous catalysis, Catalytic mechanisms

Correction to: *Nature Communications* 10.1038/s41467-024-54578-3, published online 27 November 2024

The original version of this Article omitted a line in the Acknowledgements section: Work at the Molecular Foundry was supported by the Office of Science, Office of Basic Energy Sciences, of the U.S. Department of Energy under Contract No. DE-AC02-05CH11231.

This has been now added into the last line of the acknowledgements section in both the PDF and HTML versions of the Article.

