# Supplementary material for: The role of manganese in CoMnOx catalysts for selective long-chain hydrocarbon production via Fischer-Tropsch synthesis
Source: Nat Commun. 2024 Nov 27;15:10294. doi: 10.1038/s41467-024-54578-3 (PMC11603050; doi:10.1038/s41467-024-54578-3)
Supplement: Supplementary file 1 — Supplementary Information [file 41467_2024_54578_MOESM1_ESM.pdf]

The Role of Manganese in $CoMnO_x$ Catalysts for Selective Long-Chain Hydrocarbon Production via Fischer-Tropsch Synthesis

Hao Chen[1], Zan Lian[2], Xiao Zhao[3,4], Jiawei Wan[3,4], Priscilla F. Pieters[5], Judit Oliver-Meseguer[1], Ji Yang[3], Elzbieta Pach[3], Sophie Carenco[3], Laureline Treps[3], Nikos Liakakos[3], Yu Shan[1,4], Virginia Altoe[6], Ed Wong[6], Zengqing Zhuo[7], Feipeng Yang[7], Ji Su[3], Jinghua Guo[7], Monika Blum[7], Saul Lapidus[8], Adrian Hunt[9], Iradwikanari Waluyo[9], Hirohito Ogasawara[10], Haimei Zheng[3,4], Peidong Yang[1,4,5], Alexis T. Bell[1,11], Núria López[2], Miquel Salmeron[1,3,4]*

[1]Chemical Sciences Division, Lawrence Berkeley National Laboratory, Berkeley, California 94720, United States

[2]Institute of Chemical Research of Catalonia (ICIQ-CERCA), Barcelona Institute of Science and Technology (BIST), Av. Països Catalans 16, 43007 Tarragona, Spain

[3]Materials Science Division, Lawrence Berkeley National Laboratory, Berkeley, California 94720, United States

[4]Department of Materials Science and Engineering, University of California, Berkeley, California 94720, United States

[5]Department of Chemistry, University of California, Berkeley, California 94720, United States

[6]Molecular Foundry, Lawrence Berkeley National Laboratory, Berkeley, California 94720, United States

[7]Advanced Light Source, Lawrence Berkeley National Laboratory, Berkeley, CA, 94720, United States

[8]Advanced Photon Source, Argonne National Laboratory, Lemont, IL, 60439, United States

[9] National Synchrotron Light Source II, Brookhaven National Laboratory, Upton, NY 23 11973, United States

[10] SLAC National Accelerator Laboratory, 2575 Sand Hill Road, Menlo Park, California 94025, United States

[11]Department of Chemical and Biomolecular Engineering, University of California, Berkeley, California 94720, United States

*Corresponding author. E-mail: mbsalmeron@lbl.gov

Supplementary Methods

Spin polarized density functional theory (DFT) simulations were performed using the Vienna Ab Initio Simulation Package (VASP)[1, 2]. The Perdew-Burke-Ernzerhof (PBE) functional[3] was used as the exchange-correlation functional approximation with our refitted DFT-D2 van der Waals interaction parameters[4-6]. For the valence electrons, a plane-wave basis set was adopted with an energy cutoff of 450 eV, and the ionic cores were described with the projector augmented-wave (PAW) method. The reaction pathways were simulated by using the climbing nudged elastic band (CI-NEB) method[7]. The total energy was converged to an accuracy of 1×10^{-6} eV, and a force tolerance of 0.03 eV/Å was used in all structure optimization and 0.05 eV/Å for transition state search. The description of localized 3d electrons of Mn was introduced by the approach of Dudarev et al.[8] A value of U – J = 4.0 eV was used for Mn in this study.

A 4 layer slab of p(4×4) supercell, with the bottom 2 layers fixed, is used to model the Co(0001) with k-point grid of 5×5×1 to sample the Brillouin zone. A 4 layer slab of p(2×2) supercell with bottom 2 layers fixed are used to model the MnO(100) with k-point grid of 3×3×1. We acknowledge that other Co facets may also contribute to the Fischer-Tropsch synthesis reaction. However, to specifically investigate the critical role of Mn, we simplified the structural complexity by selecting the Co(0001) facet, which has the lowest surface energy, as the starting structural model. The $CoMnO_x$ model was built by replacing two columns of $5\times2\sqrt{3}$ Co(0001) with MnO, with a mismatch along x axis ($\frac{3\cdot d_{Mn\text{-}O}-3\cdot d_{Co\text{-}Co}}{5\cdot d_{Co\text{-}Co}}$) of 6.0% and 3.9% along the y axis. The size and shape of the cell were optimized by fixing the z coordinates of the bottom two layers of Co. A K-point grid of 4×6×1 was used to sample the Brillouin zone of the $CoMnO_x$ model. The structure of the interface was decided via minima hopping simulation. Around 50 AIMD and optimization were run in the global optimization with initial thermalization temperatures of 1000 to 5000 K. In total, 26 minima were found, and the energy of the most stable one (index 24) is 0.74 eV lower than the initial structure (index 0), **Fig.S18**. The structure shows that 5 oxygen atoms stay in the interface and the other three move to the Co sites away from the interface, **Fig.S19**. Considering the stability under reaction condition (**Fig.S10**), the interface with 4 oxygen is most representative and, therefore, it is used in the reaction study.

The adsorption energies in Table S1 were calculated using CO, H_2, and H_2O as reference. As follows:

$$E_{ad}\left(C_xH_yO_z\right) = E\left(C_xH_yO_z{}^{*}\right)\text{-}[E(*)+x\cdot E(CO)+(\frac{y}{2}\text{-}z+x)\cdot E(H_2)+(z\text{-}x)\cdot E(H_2O)]$$ (**Equation_1**)

Where $E\left(C_xH_yO_z{}^{*}\right)$ is the energy of the adsorption state of species $C_xH_yO_z$ on slab, $E(*)$is the energy of slab, $E(CO)$, $E(H_2)$, and $E(H_2O)$ is the energy of isolated CO, H_2, and H_2O molecule.

Supplementary Figures

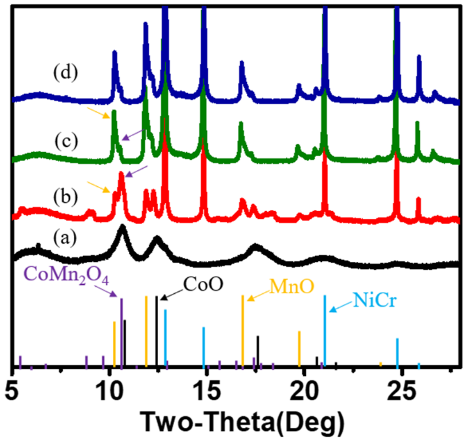

Fig.S1 In-situ X-Ray Diffraction pattern of the CoMn nanocrystals: a) as prepared; b) after oxidation at 300 ^{0}C for 2.5 h; c) after H_2 reduction at 450 ^{0}C for 2.5 h; and d) under FTS syngas conditions at 220 ^{0}C for 10 h. Bottom strips of the peaks listed in the XRD pattern of CoMnO (PDF #33-0945), CoO(PDF #74-2391), MnO(PDF #78-0424) and NiCr thermocouple, PDF # 01-1126), respectively. The wavelength of incident X-ray at Beamline 11-BM of the Advanced Photon Source at Argonne National Laboratory is 0.41 Å.

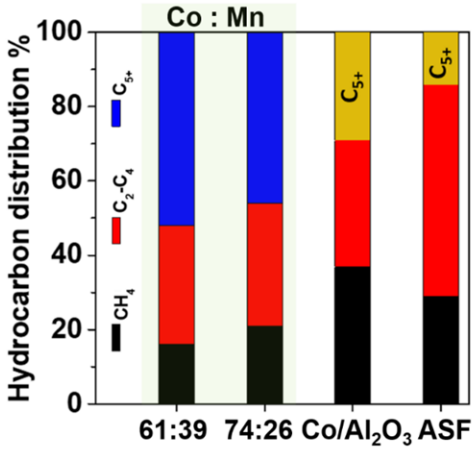

Fig.S2 Representative results of hydrocarbon products distribution for FTS reaction. Data were measured from $CoMnO_x$ nanoparticles for two Co: Mn composition ratios, and for a pure Co catalyst. The prediction from the ASF model for a chain growth probability of 0.5 is also shown. Methane product selectivity (black bars) is highest in the absence of Mn.

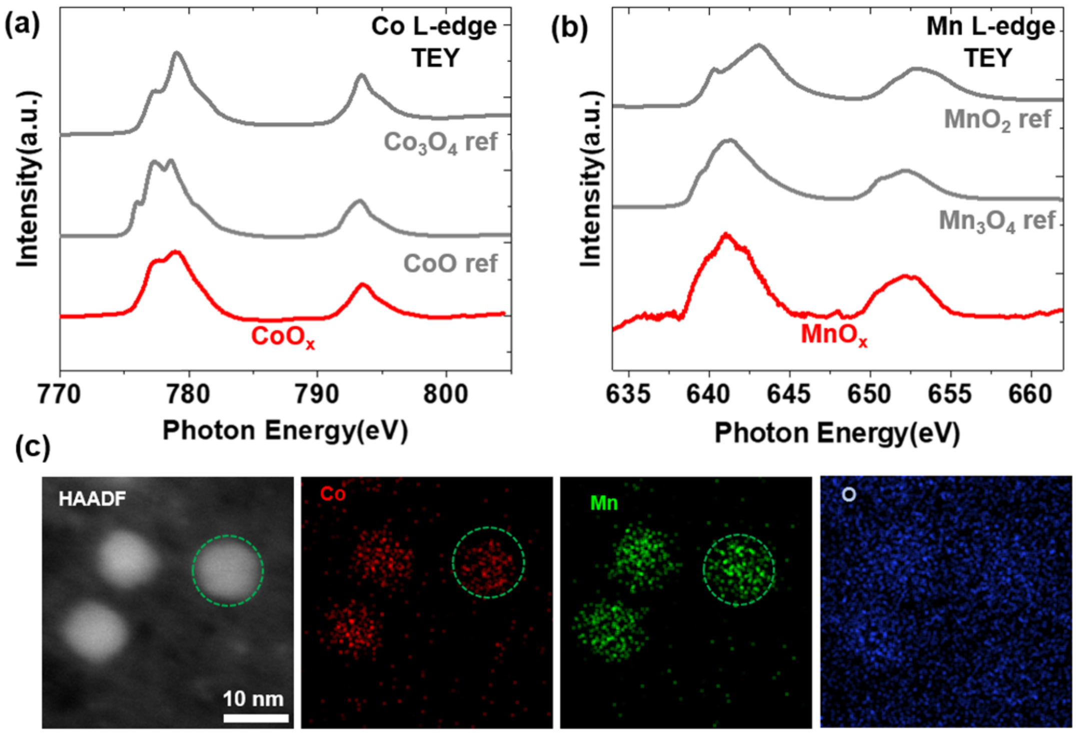

Fig.S3. (a-b) Co L-edge and Mn L-edge Total Electron Yield X-ray Adsorption Spectra (TEY-XAS) and **(c)** STEM-EDS mapping images of $CoMnO_x$ after O_2 oxidation at 300 ºC under 1 bar.

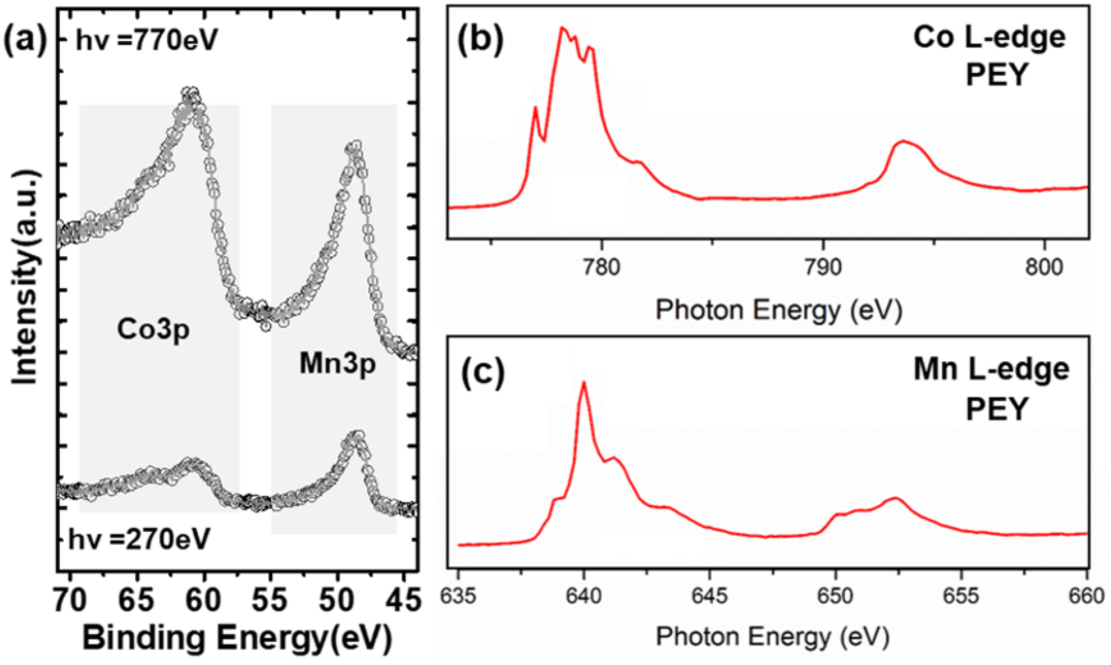

Fig.S4 (a) Depth profile XPS of $CoMnO_x$ NPs under 100 mTorr of H_2 at 300 ºC. **(b-c)** Partial electron yield X-Ray Adsorption Spectra (PEY-XAS) of Co and Mn L-edges from $CoMnO_x$ under 400 mTorr H_2 at 300 ºC. The XAS spectra were acquired with the analyzer pass energy window set at 100 eV to collect electrons with a kinetic energy of 300 eV and to suppress contributions from the gas phase[9].

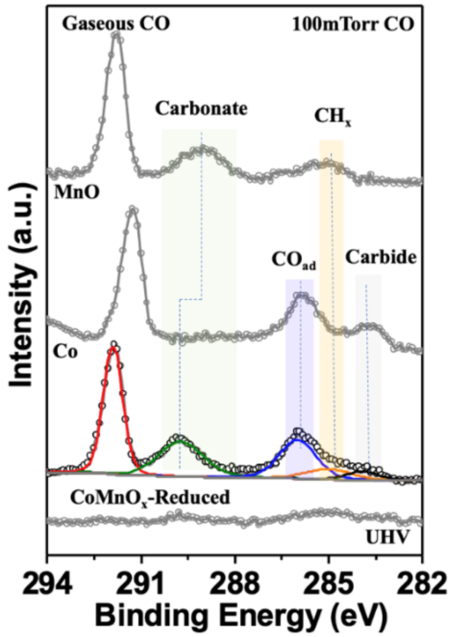

Fig.S5 C1s APXPS region of the activated $CoMnO_x$ thin film before and after room temperature (RT) exposure to 100 mTorr CO. From bottom: Spectrum in UHV after activation, showing that the $CoMnO_x$ surface is essentially clean (gray curve). Above: spectrum after introducing 100mTorr of CO, with red, green, blue, orange and grey fitting curves for CO gas phase at ~292 eV (with small changes due to different sample work function [10]), carbonate (~289.5 eV), chemisorbed CO (~286.0 eV), CH_x (~285.0 eV), and carbide (~283.5 eV) species. On pure Co, only chemisorbed CO and small amount of carbides are formed. On pure MnO (top), only peaks from carbonate and CH_x adventitious adsorbates are present, due to contamination from hydrocarbon background gases after leaking CO into the chamber[11-15]. From these results we conclude that on activated $CoMnO_x$, CO species adsorb molecularly on the Co^0 sites, and forms carbonates on MnO.

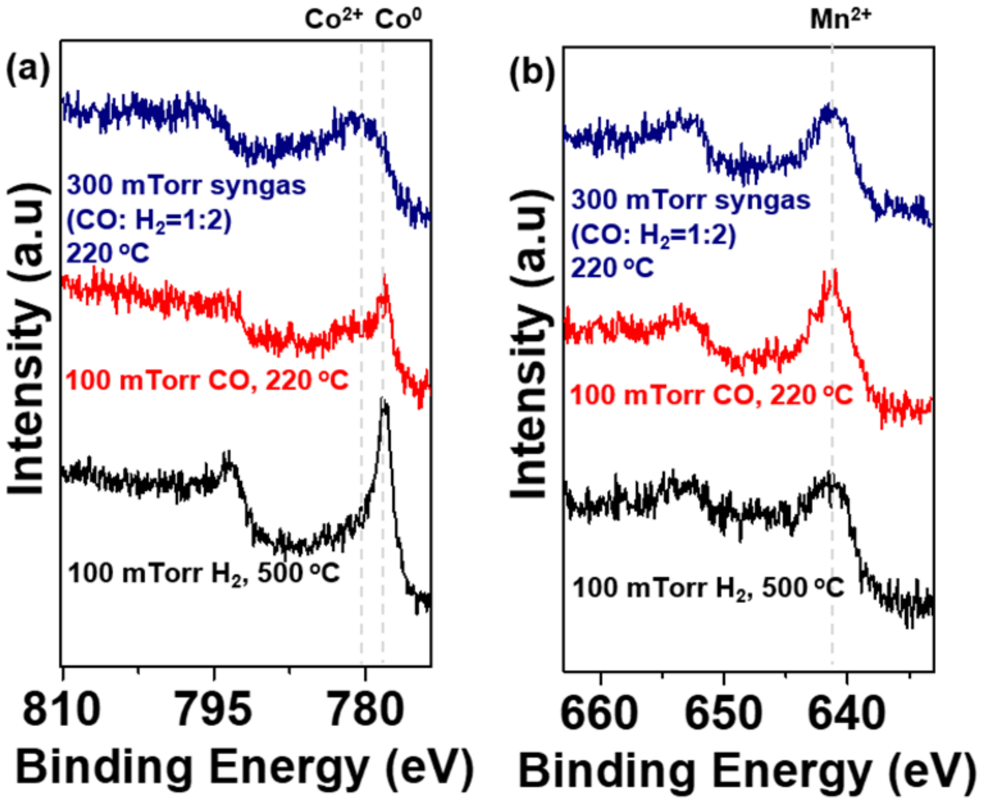

Fig.S6 APXPS from $CoMnO_x$ NPs under Fischer−Tropsch synthesis (FTS) reaction conditions. (a) Co 2p and (b) Mn 2p APXPS of $CoMnO_x$ NPs acquired sequentially under 100 mTorr H_2 at 500 ^oC (bottom, black curve), 100 mTorr CO at 220 ^oC (middle, red) and 300 mTorr syngas 220 ^oC (upper, blue). Changes in oxidation state of Co are clearly visible, while the Mn oxidation state does not change appreciably.

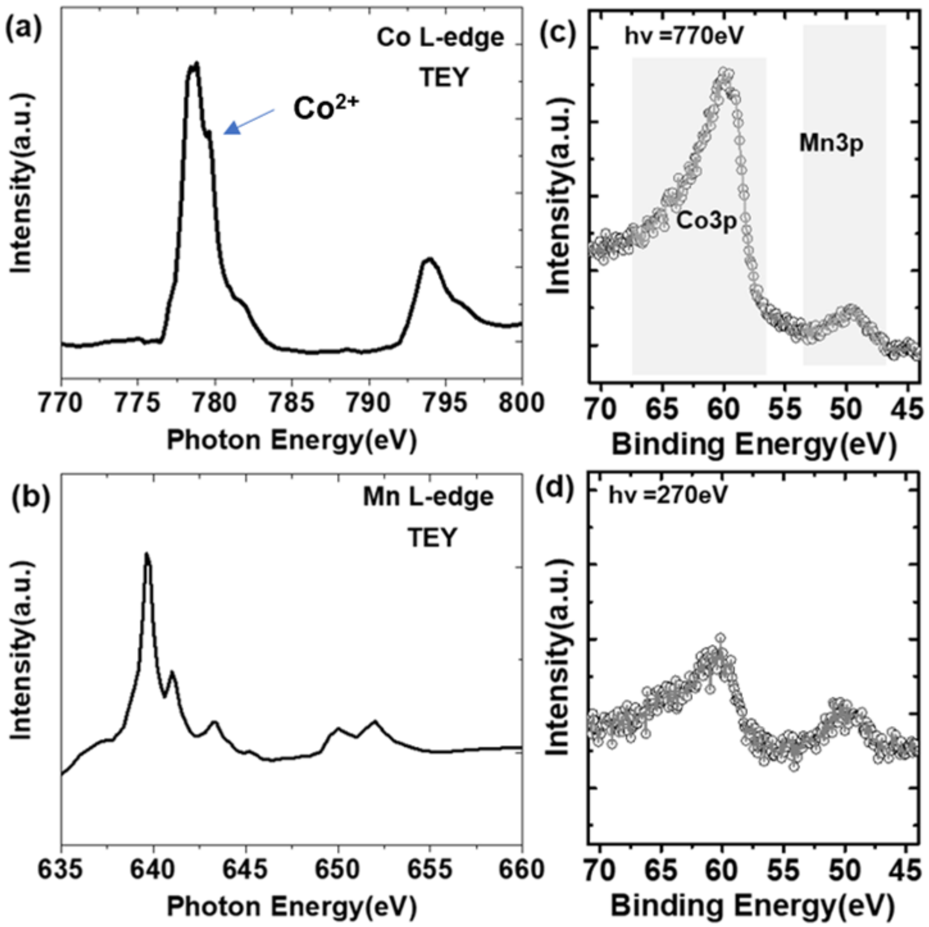

Fig.S7 (a-b) Co and Mn L-edge XAS of $CoMnO_x$ after exposure to 1 bar syngas at ~220 ºC and then measured under He. (c-d) Depth profile XPS of Co-MnO under 300 mTorr syngas ($CO:H_2$=1:2) at ~220 ºC.

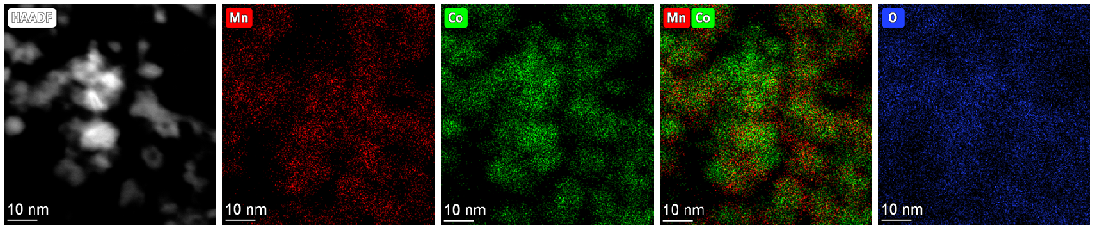

Fig.S8 $CoMnO_x$ NPs after 10 hours FTs reaction. The EDS images indicate that Co and Mn remain intimately mixed down to sub-nanometer scale.

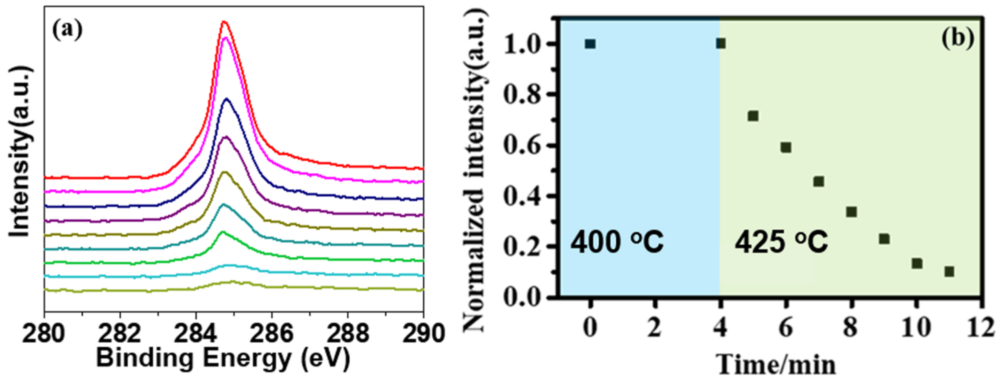

Fig.S9 (a) XPS peak intensity of CH_x species on $CoMnO_x$ surface as a function of annealing time in UHV. The first curve on top of panel (a) was measured under 400 °C, and other curves were measured at 425 °C. (b) Peak intensity vs, time.

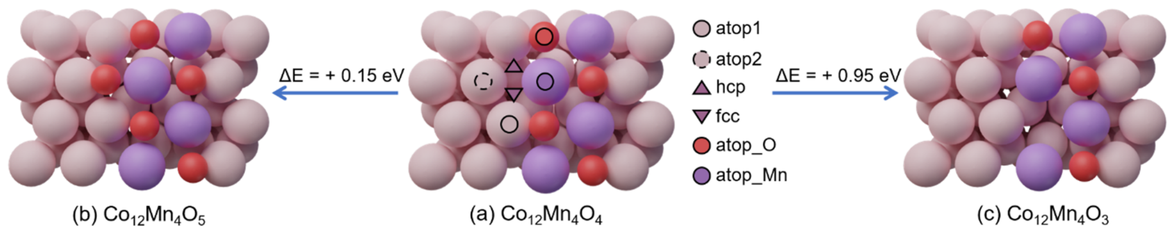

Fig. S10 Structure and stability of the MnO_x/Co(0001) model for DFT simulations. (a) Top view of a model catalyst surface containing 12 Co atoms (pink), 4 Mn atoms (purple), and 4 O atoms (red) in the topmost layer. To test the stability of this model the reaction energies with H_2 and H_2O leading to gain (b), and loss (c) of O were calculated with results shown by the arrows. The sites considered for adsorption of molecular intermediates are marked in the center panel by circles (top sites), and by triangles (3-fold hollow fcc and hcp sites).

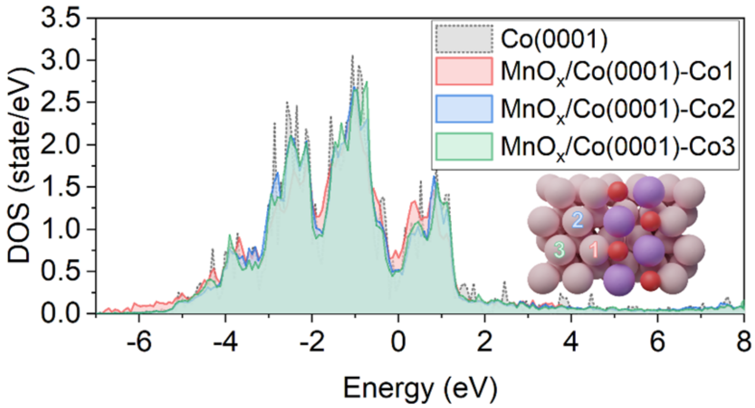

Fig.S11 Projected density of states (PDOS) of Co d-band. The insert figure shows the index of Co atom used in the PDOS plot, Co in pink, Mn in purple, O in red.

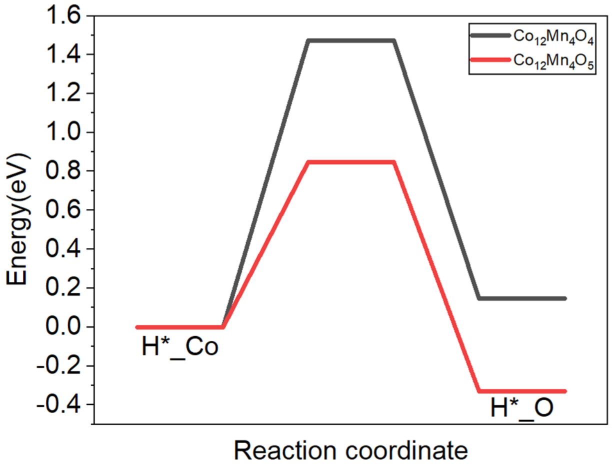

Fig.S12 Energetics of H diffusion from Co sites on $CoMnO_x$ to O sites on $CoMnO_x$. The structure of $Co_{12}Mn_4O_4$ and $Co_{12}Mn_4O_5$ is shown in **Fig.S10**.

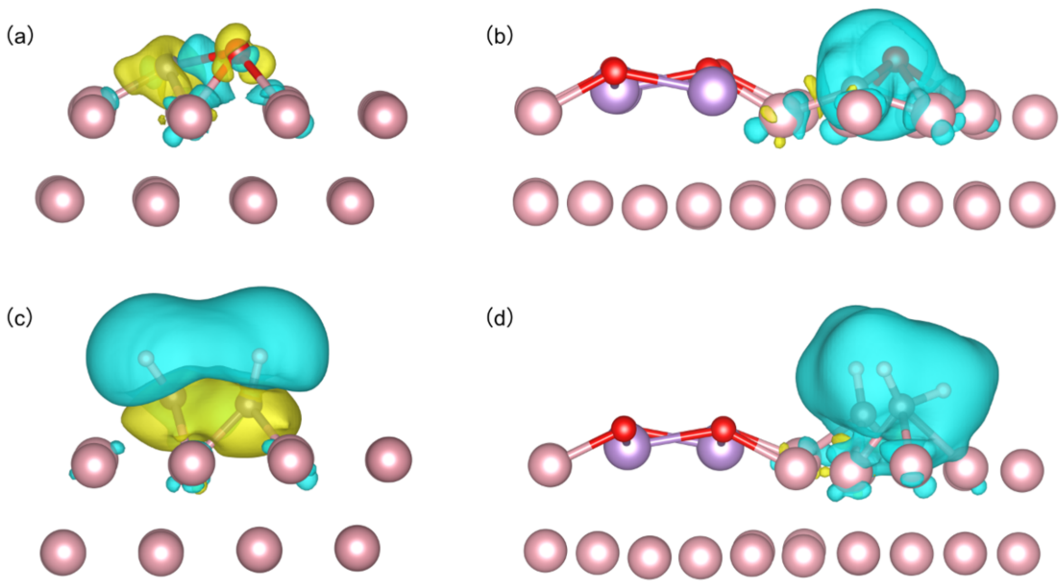

Fig.S13 Charge density difference of the transition state of (a-b) CO directly dissociation and (c-) CH* CH_2* coupling. The translucid surface shows the isosurface of 0.01 e/Bohr^3. Yellow indicates increased charge density, cyan indicates decreased charge density. Co in pink, Mn in purple, O in red, H in white, and C in gray.

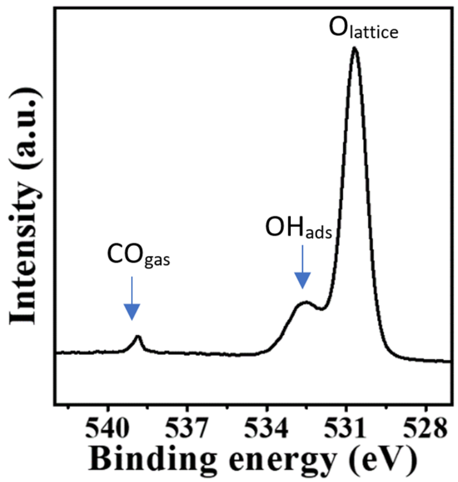

Fig.S14 O1s spectra of $CoMnO_x$ under 300 mTorr syngas ($CO:H_2$=1:2) at ~220 ºC. Lattice oxygen from $CoMnO_x$, hydroxyls, and gaseous CO are observed at ~530.7eV, ~532.5eV and~538.9 eV, respectively.

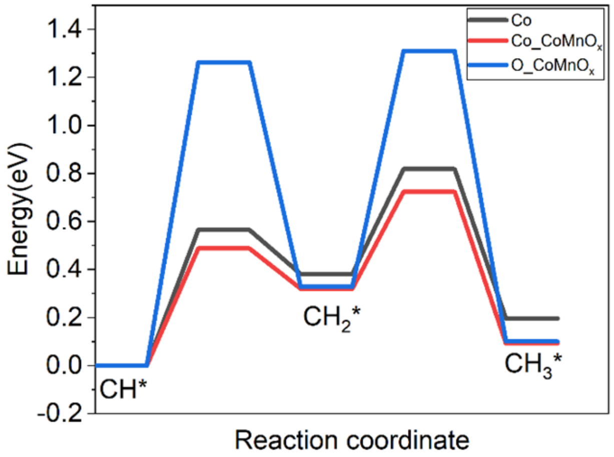

Fig.S15 Energy diagram of C-H coupling reactions on Co (0001) and Co and O sites on $CoMnO_x$. Relative energies between elementary steps were shifted to align the initial states for energy barrier comparisons. The structures of transition states are shown in **Fig.S16**.

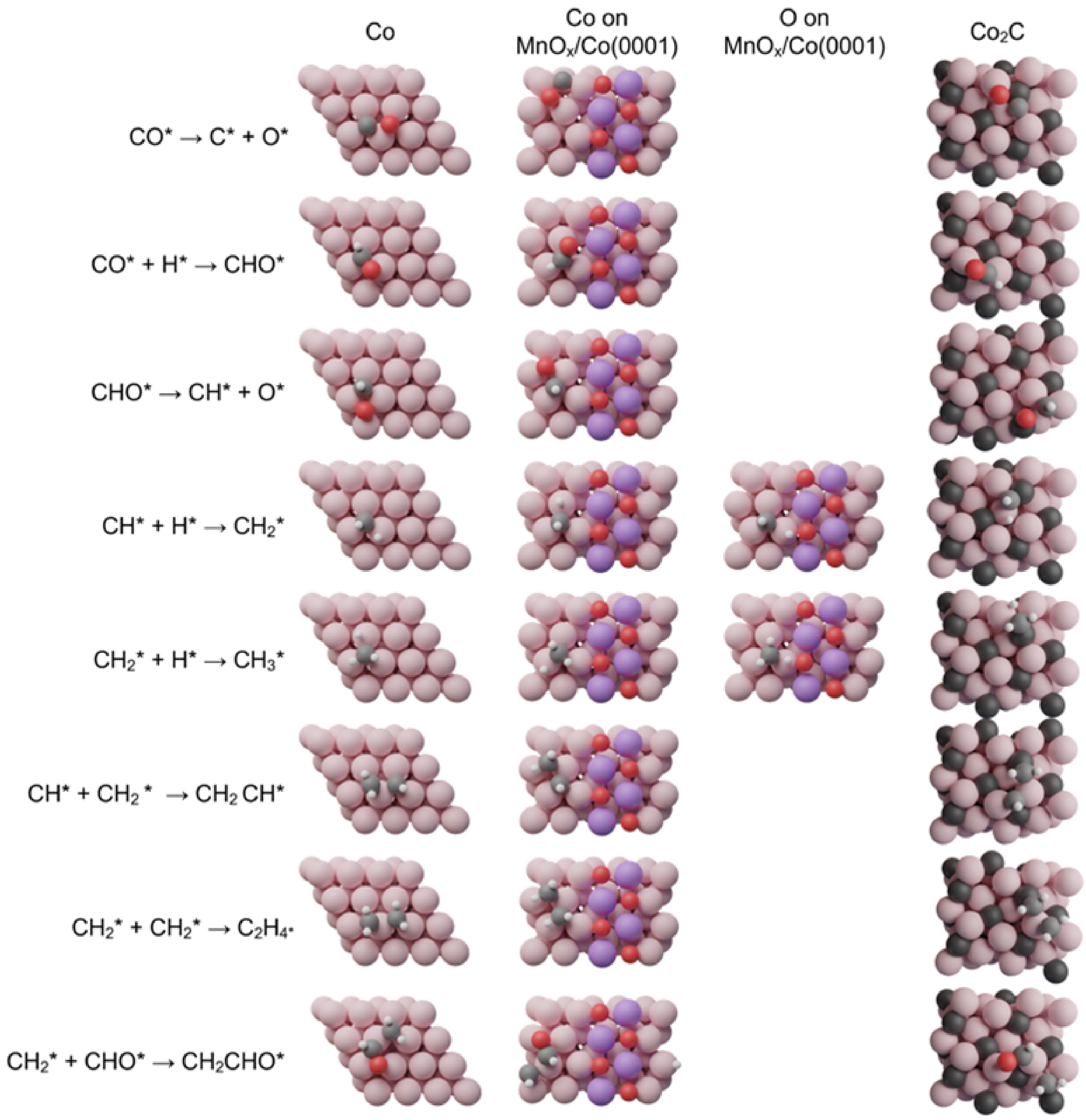

Fig.S16 The transition state of the reactions on Co, Co site on MnO_x/Co(0001), O site on MnO_x/Co(0001), and Co_2C. Co in pink, Mn in purple, C in the Co_2C slab in black, O in red, H in white, and C in adsorbates in gray.

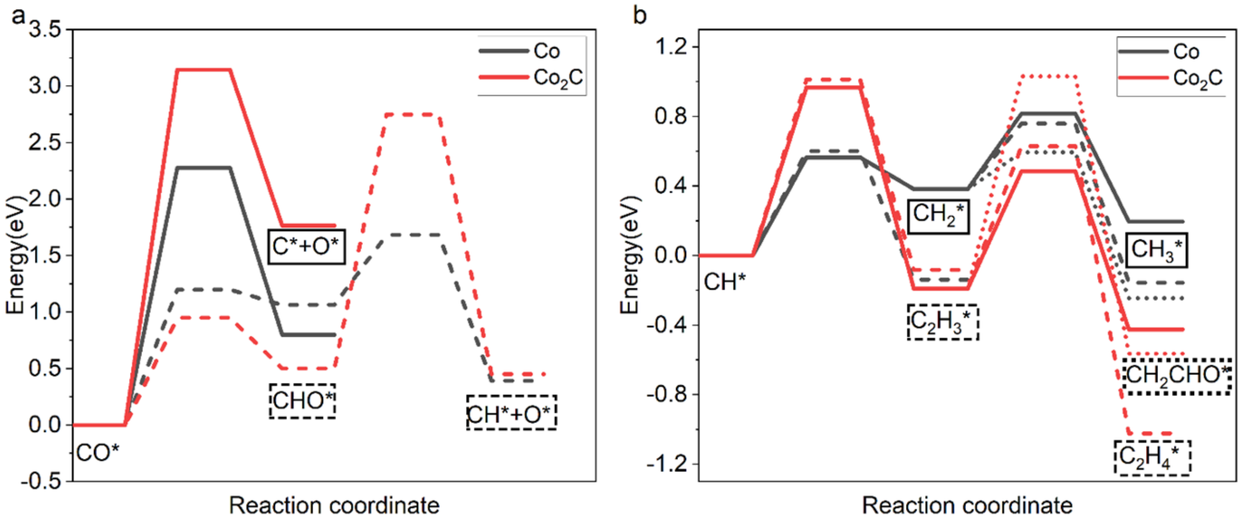

Fig.S17 Energy diagrams. (a) CO dissociation reaction: Solid lines for thermally activated process; dashed lines for H_2-assisted process; The black line for pure Co; red line for Co_2C. (b) C-H and C-C bond formation reactions: Solid lines for CH*+H* reactions. Dashed lines for CH_x*+CH_2* reactions. Dotted lines for CH_2*+CHO* reaction. Relative energies between elementary steps were shifted to align the initial state for energy barrier comparisons.

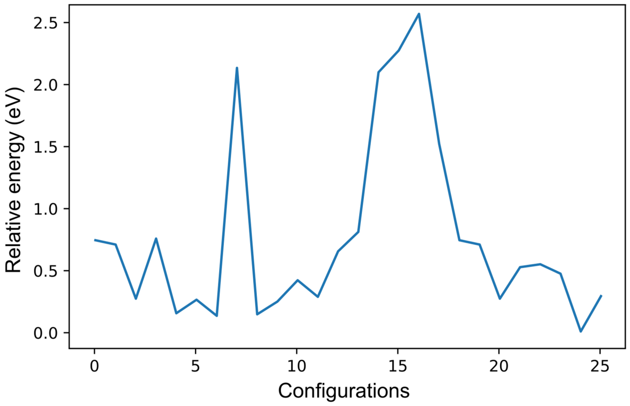

Fig.S18 Relative energy of the minima of different MnOx/Co(0001) configurations (Fig. S19) found using minima hopping algorithm.

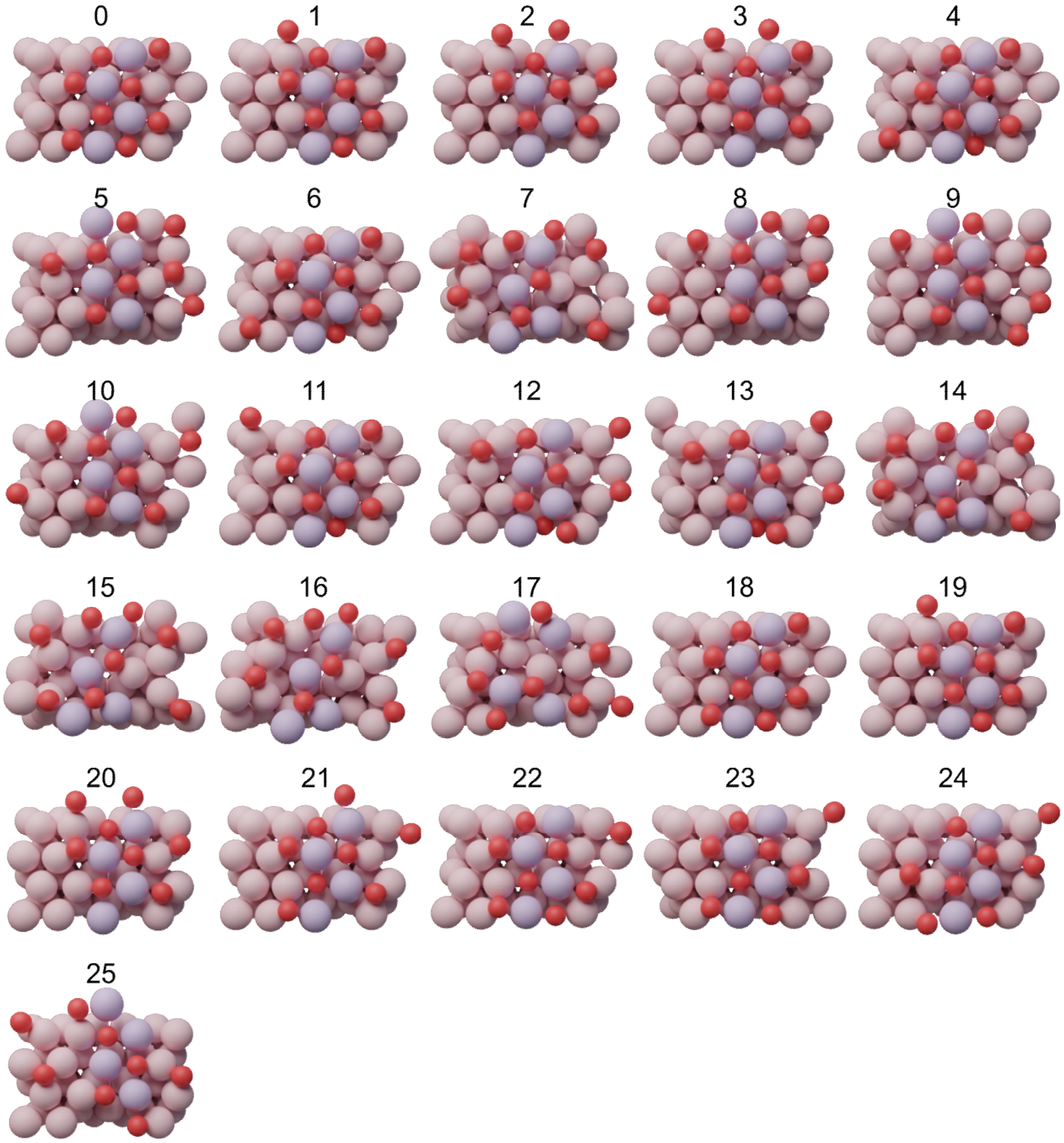

Fig.S19 The structure of the MnOx/Co(0001) model from minima hopping simulation. Co in pink, Mn in purple, O in red.

Supplementary Tables

Table S1. The adsorption of different species on sites on MnO(100), Co(0001), and MnO_x/Co(0001).
All energies computed with respect to gas-phase reservoirs of CO, H_2, and H_2O leading to both endothermic (positive) and exothermic (negative) values.

Slab		MnO		Co			MnO_x/Co(0001)					
Initial site		atop_O	atop_Mn	atop	hcp	fcc	atop1	atop2	hcp	fcc	atop_O	atop_Mn
H*	relaxation	atop_O	atop_Mn	atop	hcp	fcc	fcc_Co[a]	fcc_Co	fcc_Co	hcp_Co[b]	atop_O	atop_Mn
	E_{ad} (eV)	0.66	1.06	0.01	-0.57	-0.60	-0.54	-0.54	-0.54	-0.50	-0.39	1.33
O*	relaxation	-	-	atop	hcp	fcc	hcp_Co	brg	hcp	fcc	sub[c]	brg[d]
	E_{ad} (eV)	-	-	1.32	-0.22	-0.09	-0.22	0.15	0.10	0.12	0.64	0.15
C*	relaxation	-	-	atop	hcp	fcc	hcp_Co	brg_sub[e]	brg_Co[f]	hcp_Co	CO	brg_sub
	E_{ad} (eV)	-	-	1.26	-0.89	-0.58	-0.94	-0.95	-1.20	-0.94	1.14	-0.95
CO*	relaxation	atop_Mn	atop_Mn	atop	hcp	fcc	atop_Co[g]	atop2	atop2	atop2	atop_Mn	atop_Mn
	E_{ad} (eV)	-0.01	-0.01	-1.63	-1.65	-1.63	-1.66	-1.66	-1.66	-1.66	-0.06	-0.06
CH*	relaxation	atop_O	atop_Mn	atop	hcp	fcc	hcp_Co	brg_Co	fcc_Co	hcp_Co	CHO[h]	fcc_Co
	E_{ad} (eV)	0.77	2.63	-1.60	-1.77	-1.60	-1.71	-1.60	-1.52	-1.71	-0.23	-1.51
CH_2*	relaxation	atop_O	atop_Mn	atop	hcp	fcc	hcp_Co	fcc_Co	fcc_Co	hcp_Co	atop_O	fcc_Co
	E_{ad} (eV)	-0.14	0.56	-0.99	-2.00	-1.94	-1.93	-1.86	-1.86	-1.93	-0.48	-1.86
C_2H_4*	relaxation	-	-	atop	hcp	fcc	atop1	atop2	atop2	atop2	Phy[i]	phy
	E_{ad} (eV)	-	-	-4.39	-4.52	-4.52	-4.17	-4.47	-4.47	-4.47	-3.41	-3.62

[a] The fcc site on Co surface in the MnO_x/Co(0001) model.
[b] The hcp site on Co surface in the MnO_x/Co(0001) model.
[c] The subsurface under MnO_x in the MnO_x/Co(0001) model.
[d] The bridge site between atop2 and atop_Mn in the MnO_x/Co(0001) model.
[e] The subsurface bridge site between atop2 and atop_Mn in the MnO_x/Co(0001) model.
[f] The bridge site on Co surface in the MnO_x/Co(0001) model.
[g] The atop site on Co surface in the MnO_x/Co(0001) model.
[h] The O in MnOx move to the surface and format CHO* with CH*.
[i] Physisorption.

Table S2. Activation and reaction energies, E_a and ΔE in eV, for all the FTs elementary steps investigated on the Co, MnO_x/Co(0001) and Co_2C systems.

Reaction	Co		Co on MnO_x/ Co(0001)		O on MnO_x/ Co(0001)		Co_2C	
	E_a	ΔE	E_a	ΔE	E_a	ΔE	E_a	ΔE
CO* → C* + O*	2.28	0.80	2.02	0.40	-	-	3.15	1.77
CO* + H* → CHO*	1.20	1.06	0.98	0.98	-	-	0.95	0.50
CHO* → CH* + O*	0.62	-0.67	0.58	-0.69	-	-	2.25	-0.05
CH* + H* → CH_2*	0.56	0.38	0.49	0.32	1.26	0.33	0.82	-0.83
CH_2*+ H* → CH_3*	0.44	-0.19	0.40	-0.23	0.98	-0.23	0.97	-0.19
CH* + CH_2*→ CH_2CH*	0.60	-0.14	0.56	-0.08	-	-	0.68	-0.23
CH_2*+ CH_2*→ C_2H_4*	0.38	-0.54	0.36	-0.61	-	-	1.01	-0.08
CH_2* + CHO* → CH_2CHO*	0.21	-0.63	0.22	-0.67	-	-	1.22	-0.37

Supplementary References

1. Kresse, G. and J. Furthmuller, *Efficient iterative schemes for ab initio total-energy calculations using a plane-wave basis set.* Physical Review B, 1996. **54**(16): p. 11169-11186.
2. Kresse, G. and J. Furthmuller, *Efficiency of ab-initio total energy calculations for metals and semiconductors using a plane-wave basis set.* Computational Materials Science, 1996. **6**(1): p. 15-50.
3. Perdew, J.P., K. Burke, and M. Ernzerhof, *Generalized gradient approximation made simple.* Physical Review Letters, 1996. **77**(18): p. 3865-3868.
4. Grimme, S., *Semiempirical GGA-type density functional constructed with a long-range dispersion correction.* Journal of Computational Chemistry, 2006. **27**(15): p. 1787-1799.
5. Bučko, T., et al., *Improved Description of the Structure of Molecular and Layered Crystals: Ab Initio DFT Calculations with van der Waals Corrections.* The Journal of Physical Chemistry A, 2010. **114**(43): p. 11814-11824.
6. Almora-Barrios, N., et al., *Costless Derivation of Dispersion Coefficients for Metal Surfaces.* Journal of Chemical Theory and Computation, 2014. **10**(11): p. 5002-5009.
7. Henkelman, G., B.P. Uberuaga, and H. Jónsson, *A climbing image nudged elastic band method for finding saddle points and minimum energy paths.* The Journal of Chemical Physics, 2000. **113**(22): p. 9901-9904.
8. Dudarev, S.L., et al., *Electron-energy-loss spectra and the structural stability of nickel oxide: An LSDA+U study.* Physical Review B, 1998. **57**(3): p. 1505-1509.
9. Eren, B., et al., *Catalyst Chemical State during CO Oxidation Reaction on Cu(111) Studied with Ambient-Pressure X-ray Photoelectron Spectroscopy and Near Edge X-ray Adsorption Fine Structure Spectroscopy.* J Am Chem Soc, 2015. **137**(34): p. 11186-90.
10. Wu, C.H., et al., *Ambient-Pressure X-ray Photoelectron Spectroscopy Study of Cobalt Foil Model Catalyst under CO, H2, and Their Mixtures.* ACS Catalysis, 2017. **7**(2): p. 1150-1157.
11. Chen, H., et al., *Elucidating the active phases of CoO(x) films on Au(111) in the CO oxidation reaction.* Nat Commun, 2023. **14**(1): p. 6889.
12. Kersell, H., et al., *CO Oxidation Mechanisms on CoO(x)-Pt Thin Films.* J Am Chem Soc, 2020. **142**(18): p. 8312-8322.
13. Shirley, D.A., *Esca.* Advances in chemical physics, 1973. **23**: p. 85-159.
14. Hantsche, H., *High resolution XPS of organic polymers, the scienta ESCA300 database. By G. Beamson and D. Briggs, Wiley, Chichester 1992, 295 pp., hardcover, £ 65.00, ISBN 0-471-93592-1.* Advanced Materials, 2004. **5**(10): p. 778-778.
15. Barr, T.L. and S. Seal, *Nature of the use of adventitious carbon as a binding energy standard.* Journal of Vacuum Science & Technology A: Vacuum, Surfaces, and Films, 1995. **13**(3): p. 1239-1246.
